# Exercise Effects on the Biomechanical Properties of the Achilles Tendon—A Narrative Review

**DOI:** 10.3390/biology11020172

**Published:** 2022-01-21

**Authors:** Changxiao Yu, Liqin Deng, Li Li, Xini Zhang, Weijie Fu

**Affiliations:** 1 Key Laboratory of Exercise and Health Sciences of Ministry of Education, School of Kinesiology, Shanghai University of Sport, Shanghai 200438, China; yuchangxiao1014@163.com (C.Y.); 1921516007@sus.edu.cn (L.D.); 2 Department of Health Sciences and Kinesiology, Georgia Southern University, Statesboro, GA 30460, USA; lili@georgiasouthern.edu; 3 Shanghai Frontiers Science Research Base of Exercise and Metabolic Health, School of Kinesiology, Shanghai University of Sport, Shanghai 200438, China

**Keywords:** exercise, Achilles tendon, biomechanical properties, adaptation

## Abstract

**Simple Summary:**

The Achilles tendon influences the running economy because of its ability to store and release strain energy, and it remains one of the most vulnerable tendons among athletes and recreational runners. Exercised-related mechanical loading appears to induce changes in the Achilles tendon morphology and mechanical material properties. Both acute and relatively long-term exercise induces tendon adaptation, although biomechanical changes, e.g., cross-sectional area, plantarflexion moment, Young’s modulus, and stiffness, in response to exercise duration, type, and loading-regimes differ widely. Furthermore, a strong Achilles tendon can be developed by chronic exposure to habitual mechanical loading from daily exercise, which is associated with greater energy storage, release and overall health.

**Abstract:**

The morphological and mechanical properties (e.g., stiffness, stress, and force) of the Achilles tendon (AT) are generally associated with its tendinosis and ruptures, particularly amongst runners. Interest in potential approaches to reduce or prevent the risk of AT injuries has grown exponentially as tendon mechanics have been efficiently improving. The following review aims to discuss the effect of different types of exercise on the AT properties. In this review article, we review literature showing the possibility to influence the mechanical properties of the AT from the perspective of acute exercise and long-term training interventions, and we discuss the reasons for inconsistent results. Finally, we review the role of the habitual state in the AT properties. The findings of the included studies suggest that physical exercise could efficiently improve the AT mechanical properties. In particular, relatively long-term and low-intensity eccentric training may be a useful adjunct to enhance the mechanical loading of the AT.

## 1. Introduction

Modern human morphology has been proposed to reflect numerous features related to the evolution, from walking to running, that occurred two million years ago [1]. Along with features such as larger joint surfaces, shorter toes, and a medial longitudinal plantar arch, the Achilles tendon (AT) lengthened significantly. This is significant as the tendon stores significant elastic strain energy during the initial stance phase of running and then releases the energy through recoil during the subsequent propulsion [2,3]. These reports suggested that the external mechanical loads can be adapted by adjusting AT elastic modulus, stiffness, and size based on the nature structure [4]. As the strongest and thickest tendon in the human body, the mechanical loading of the AT is great because of the large internal plantar flexor moment that occurs at the ankle during the stance phase of running [5]. Previous studies have shown that the AT can load up to four times the body’s weight when walking, and 12.5 times the body’s weight when running and jumping [6,7,8].

As one of the most common sports injuries, Achilles tendinopathy accounts for 8–15% of all running injuries [9], with a high lifetime risk of 52% among elite male runners [10]. This injury will compromise sports performance, may prevent active, athletic individuals from participating in physical activities, can interfere with daily living, and may possibly lead to tendon rupture [11,12]. Unfortunately, conservative treatment is not always successful and can lead to surgical intervention (25–45% of patients) [13], with a cost ranging from $10,000 to as much as $31,000 (https://www.howmuchisit.org/achilles-tendon-surgery-cost/) (accessed on 26 November 2021). In 3–5% of cases, it can be career-ending for athletes, resulting in a health and personal cost both immediately and in the future [7]. From recreational to elite athletes, the reduction in physical activity can result in overall physical deconditioning and can increase the risk of cardiovascular problems in time [14,15,16].

Achilles tendinopathy causes pain, swelling, and thickening in the AT, and ultimately impairs health-promoting activities [17]. It most often occurs in the mid-substance of the tendon (55–65%) and less commonly at the insertion (20–25%) [18]. The cause is not fully understood but is essentially a maladaptive response to increased or excessive loading that leads to progressive weakening and dysfunction of the specific tendon area and then to disrepair and degenerative changes [7]. Previous Delphi studies [11,12] identified several factors related to Achilles tendinopathy in an active, athletic population. These included previous tendinopathy or injuries, advancing age, sex, and antibiotic treatment. Moreover, training errors, hard running surfaces, poor ankle strength and flexibility are risk factors for inducing Achilles tendinopathy [19]. It is worth noting that ultrasound is both specific and sensitive enough for assessing the tendon structure, especially for the diagnosis of Achilles tendinopathy [20,21]. Based on the ultrasound imaging technology, a more recent study found that the cross-sectional area (CSA) of the AT could have the potential to be an effective method for assessing the risk of high Achilles tendinopathy based on the correlation between the CSA and height, sex, height, miles run per week, and BMI [5]. 

Considering that there are modifiable (e.g., running distance, duration) and unmodifiable (e.g., age, sex) factors affecting the AT injuries, prevention strategies need to be proposed and used. Both concentric and eccentric strengthening training have been advocated for improving AT function in the past [22,23,24]. Alfredson et al. [23] found that 12 weeks of eccentric training for the calf muscle could significantly reduce pain during running activity and increase the muscle strength for patients with chronic Achilles tendinosis. Another study supports that this training method may improve clinical results for these patients [24]. However, more recently, both isometric and functional strengthening have been promoted. The purpose of this narrative review is to examine the effect of different exercise regimens on the human AT. We begin by reviewing the acute effect of different types of exercise on the tendon. We then assess the effect of differing training regimens on the AT properties. This would be followed by a review of how different habituated states influence the tendon. In the end, we identify gaps in our knowledge and make suggestions for future directions. The overall aim of this paper is to increase awareness of the optimal ways for sports enthusiasts to enhance their AT properties and maintain their healthy state to reduce injury risk.

## 2. Methods

In this narrative review, databases including PubMed, EBSCOhost, Scopus, Web of Science, and Google Scholar from October 1960 to October 2021 were searched for the following terms: “Achilles tendon” OR “AT” AND “exercise” OR “training” AND “mechanical properties” OR “material properties”. All articles searched in the databases were imported into the EndNote (EndNote X7, Stanford, CA, USA). Then, full publications and abstracts were screened and all relevant papers retrieved. The search was restricted to full-text accounts written in English. Following this process, the authors manually searched the reference lists of all selected papers and used the “cited by” feature available in three of the databases to check for additional papers not found in the initial search.

To limit the number of included studies, papers considered for this review needed to satisfy one of the following inclusion criteria: (1) address the effect of acute exercise on human AT mechanical properties; (2) address the effect of long-term training on changes in human AT mechanical properties; (3) address the effect of a habitual state (sports activity, footwear, etc.) on the human AT properties. In addition, the participants must be healthy adults without any musculoskeletal injury or psychiatric disorders. All these procedures were in line with published guidelines for writing a narrative review [25].

**Figure 1:**
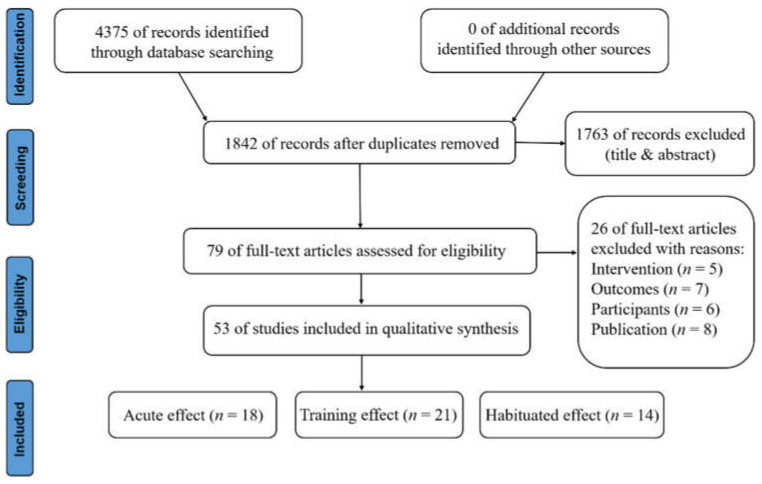
PRISMA flowchart of the narrative review.

As shown in Figure 1, the selection process is visualized in the PRISMA flow diagram. The electronic search yielded a total of 4375 studies, of which 53 (acute effect, 18; training effect, 21; habituated effect, 14) were included. After the removal of duplicates, 1842 studies were manually reviewed. A total of 1763 studies were excluded after assessing title and abstract review, then full-text articles of the remaining 79 studies were screened based on the eligibility criteria.

## 3. Practical Considerations

### 3.1. Effects of Acute Exercise on the Achilles Tendon

A summary of the characteristics of five studies examining the AT biomechanical response to the acute interventions can be found in Table 1. Previous studies have indicated that adaptive changes in the mechanical properties of connective tissues, such as the AT, are affected by external loads [26,27]. The average peak strain in the AT during walking, running, and hopping were 4.6%, 5.8%, and 8.3%, respectively [28,29]. In addition, these studies also found that the AT has high compliance under large external forces, and its length changes considerably during movement. The external mechanical load imposed externally to fibrous connective tissues could influence the length and stretching velocity of the tendons, increasing the high compliance of the AT [30]. Previous investigations have shown that the low braking forces, large vertical and propulsive forces [2], and increased internal force [4] of the triceps surae during the stance phase of running are significantly related to reduced AT injuries, given the superior mechanical and material properties of the AT [31] and the basic factors of AT injury prevention.

Changes in tendon mechanical behavior, e.g., an increase in tendon compliance, have been shown to occur in response to acute intense exercise. A systematic review confirmed that the mechanical and morphological properties of the AT were affected by acute exercise patterns [36]. Previous studies suggested that for the AT specificity, it would be valuable to know the time taken to return to baseline values in women compared to men, which may explain the cause of the lower AT injury rates among women [31,37,38]. For instance, passive stretching decreased stiffness of the medial gastrocnemius tendon acutely, with women demonstrating a far greater increase in compliance (22.4%) compared with men (8.8%) after 5 min of a passive dorsiflexion stretch [37]. It may be due to the woman’s tendon being less stiff at baseline before intervention based on the relevant studies [38,39]. An increase in tendon compliance responds to an acute loading protocol, however, when this acute response to load returns to baseline is not yet confirmed. The study of Zhou et al. [40] reported that the distal, middle, and proximal regions of the AT stiffness increased by 19.53%, 17.01%, and 25.73% after acute 5-minute static stretching, respectively. From the perspective of mechanical properties, these results suggest that the AT has high compliance through acute static stretching intervention, but that the tendon architecture was independent of changes in tendon stiffness [40].

Isometric plantarflexion yields an immediate decrease in stiffness that quickly plateaus with the addition of continued activity. Ten 4-s isometric plantarflexion contractions resulted in decreased tendon stiffness after the first five contractions but did not increase significantly thereafter in a sample of six men [34]. Similarly, a decreased tendon stiffness (−10.9%) was observed after six 8-s MVIC of the plantar flexors with no further decrease after an additional three 60-s of static stretching in eight men and women [33]. The stiffness of the AT decreased in non-stretch-shortening circles (SSC) training, such as isometrics contraction and static stretching training, but did not significantly change in SSC training, such as running and hopping performances [32]. This result is similar to the results of in vivo animal experiments and suggests that the SSC effect is negligible in response to tendon elongation in real life [41,42]. Furthermore, the mechanical behavior of tendons that occurs in response to acute stimuli seems to have gender-specific effects. In general, the tendons of women are less stiff at baseline before exercise intervention, namely, the higher viscoelastic properties of tendon structures in women, which means that women have a higher compliance compared to men [38]. One previous study investigated the effect of the gender factor on the AT mechanical characteristics and metrical properties during 100 toe jumps with 20% body mass, and reported the increased elongation (+32.2%), reduced AT stiffness (−30.3%) and reduced Young’s modulus (−32.1%) after that isometrics contraction exercise only in women; these responses for women may be a protective mechanism against large loads in acute exercise [31]. These results, along with the above-mentioned ones [37,38], indicated that a female’s tendon might be more compliant in response to an intense bout of loading, which might effectively reduce the risk of AT injuries and, in part, explain the large discrepancy in tendon disorders and rupture rates between genders. The hormonal differences between genders have been indicated to affect tendonous tissue, as estrogen has previously been shown to inhibit collagen synthesis, and thus affects tendon tissue properties [43]. Thus, the fact that the tendon collagen fractional synthesis rate is lower in women could, therefore, be responsible for the increased elongation and reduced stiffness of the AT [37]. However, the more specific mechanisms that cause the differences in mechanical properties of the AT between genders are unknown. This needs to be further discussed in future studies.

On the other hand, past studies have suggested that the properties of the AT could be affected by running strike patterns [6,44,45]. The plantarflexion torque exerted to resist dorsiflexion torque during running with a forefoot strike pattern was larger than that exerted to resist dorsiflexion torque during running with a rearfoot strike pattern and is important for energy release and absorption by plantar flexors [46]. Lyght et al. [6] reported that the strain and stress associated with a rearfoot strike pattern were lower than those associated with a forefoot strike pattern because the large knee flexion angle can be attributed to the short distance between the center of mass and the heel. A spring-like running posture is beneficial for shock absorption. Therefore, the authors suggested that the rearfoot strike pattern can reduce AT strain, stress, and strain rate compared to the forefoot strike pattern. Besides, increasing the stepping frequency by 5%, namely shortening the strike length, can reduce the peak AT stress and strain at a fixed speed of 3.5 m/s, regardless of foot strike pattern [6]. The muscle-tendon biomechanical differences of plantar flexors between the forefoot and rearfoot striking have been taken into account. Yong et al. [45] found forefoot striking can effectively reduce tendon energy storage of the soleus and increase the gastrocnemius muscle activation compared to the rearfoot striking running pattern. These results demonstrated that the increased eccentric contraction from the progressive strengthening plantar flexors program can reduce the risk of injuries during forefoot striking. However, Kubo et al. [47] failed to find significant differences after investigating the relationship between foot strike patterns and the AT properties in long-distance runners. Therefore, the properties of AT are largely affected by acute changes in running strike patterns. Relationships among internal mechanisms should be studied in the future. 

Existing studies have ascribed reduced AT stiffness to a prolonged run [48], different running strike patterns, or non-SSC training. Decreased stiffness could instantly increase the risk of AT injuries and affect the properties of the AT complex. SSC training does not exert an acute effect on AT properties [49]. Nevertheless, the acute effect of exercise intensity on the mechanical properties of AT remains unclear. Additionally, whether changes originate from muscle or the AT itself requires exploration.

### 3.2. Effects of Training Effects on the Achilles Tendon

Table 2 describes the studies relevant to this section. Although training has an acute effect on the AT, over-exercising presents a high risk of injuries. Present studies emphasized the adaptive potential of tendons to increased mechanical loading applied repetitively and statically, which was consistently shown despite the variety of long-term training protocols [50,51]. Arampatzis’ group found that after 14 weeks of isometric (rep) with 90% MVC exercise intervention, an increase in (1) tendon-aponeurosis stiffness [30,52,53], (2) tendon elastic modulus [30,52,53], (3) AT CSA [30,52,53], and (4) plantarflexion moment (PF) and tendon force [54] were found at high strain magnitude (high strain) with a low strain frequency (low reps). In addition, Fletcher et al. [55] did find an increase in the AT stiffness (+18.6%) and PF moment (+21.6%) after 20 s isometric (static) training with 80% MVC. It is, however, noteworthy that the training week (8w) was reduced along with only one repetition. A most recent study [50] also reported the adaptive change in the AT properties (AT stiffness: +36.1% and CSA: +7.8%) following low-load resistance training (20–35% of 1RM) with partial blood flow restriction.

In contrast, the effect of plyometric training on tendon properties still seems ambiguous [59], since the six plyometric training interventions [53,60,61,62,63,64] reported controversial results. The changes in tendon stiffness and Young’s module ranged from +19.4% [64] to −9.4% [63], and +23.9% [53] to −19.2% [63]. However, only the 27% increase in AT stiffness reported by Fouré et al. [60,61,62] reached statistical significance. The different jumping exercises, uncontrolled or comparably low (40% repetition maximum [64]) tendon load magnitude and dissimilar intervention durations (8 to 14 weeks) might be the reason for the inhomogeneous findings. Comparing dynamic (concentric-eccentric) and isometric training with plyometric training, Kubo et al. [49,64] and Bohm et al. [53] reported a statistically significant increase of the AT stiffness solely following the dynamic and isometric but not after the plyometric training.

Overall, since only four studies using running as the type of training have been involved, we cannot give a preliminary conclusion. One study’s [56] aim was to investigate the effect of habitual exercise on the structural properties of the AT in sedentary participants, the results showed that a total training stimulus of approx. 9 months of running (30–50 min habitual running for 2–3 times per week) in previously untrained subjects did not result in any significant changes in AT stiffness (+7.3%) and CSA (−0.3%). Meanwhile, another study [57] was to determine the effect of transition of running style on the AT mechanical properties over a 12-week intervention, its results seem to demonstrate an increase in AT properties (stiffness, module, CSA) in men after a 12-week minimalist running transition program and 6-month follow-up. The viscoelastic property of the AT plays a crucial role in improving stiffness and running economy while transitioning to the forefoot strike pattern given the shortened contact time and contracting velocity of plantar flexors [57]. In agreement with the recent findings, Zhang et al. [51] investigated the effect of 12-week transition training on the AT loading; they found the peak AT force could significantly increase for habitual rearfoot strikers when they were switching to the forefoot strike pattern with minimalist shoes compared with the control group who could choose the strike pattern after 12-week transition training. In addition, a study reported that after 6 months of repeated 2 km runs, as one type of rigorous endurance training program, the new elite infantries had a significant increase in the AT CSA [58] (see Table 2).

Lastly, regarding the duration of the exercise intervention, most of the above-mentioned studies featuring an exercise duration of 12–14 weeks found significant adaptations of AT properties [30,52,53,57,64,65,66], indicating that tendons already respond to increased mechanical loading within 3 months. However, one study [49] showed that AT stiffness increased significantly by 18.8 ± 10.4% for 8 weeks (4 days/w) con-ecc weight (reps) training with 70% RM. These results suggested that resistance training can effectively improve the stiffness of the tendon structures, as well as the strength and size of the muscles [49]. What is more, another more recent study [67] showed that AT stiffness and Young’s modulus improved by 25.0% (*p* = 0.004; ES = 1.73; CI 95% = 0.85–2.52) and 20.1% (*p* < 0.044; ES = 1.31; CI 95% = 0.49–2.06) after 4 weeks of high-load voluntary plantarflexion training, respectively; meanwhile a significant increased CSA (+14.7%) was observed after 8 weeks and contributed to a further increase in the AT stiffness (+62.1%) and Young’s modulus (+26.1%). The authors reported that the increased tendon stiffness may be due to the adaptations in the AT properties after 4-week high-load training. In agreement with the speculation, a literature review confirmed that the changes in tendon stiffness caused by the training intervention seem to be attributed more to the adaptability of the AT material than to the morphological properties [68].

Collectively, the AT can experience positive biomechanical adaptation when exposed to mechanical loading within a specific training volume. However, such AT strain depends on many factors known to differ between individuals [69] and seems to have a preferred strain limit to maintain the increase in triceps surae muscle strength during muscle strength training [70]. Further works are warranted to elucidate the specific mechanical loading conditions (e.g., magnitude, duration, rate, frequency) by ensuring that AT strains occur within the optimal range that elicit maximal positive (e.g., anabolic) adaptation.

### 3.3. Effects of Habituated States on the Achilles Tendon

As shown in Table 3, the differences in the AT properties (stiffness, Young’s Modulus, CSA, and strength) between runners and non-runners are obvious. To enhance mechanical properties of the AT (e.g., higher tendon stiffness, larger CSA, and Young’s modulus), a variety of habitual physical activities and exercises have been adopted [27,71,72,73]. Current studies posited that the homeostasis of connective tissues, such as tendons and ligaments, were affected by the mechanism of force transmission due to cyclic strain, which adjusted the adaptive progression and feedback of the AT during exercise under regulation by mechanical stimulus [27,71,72,73].

The influence of mechanical loading associated with exercise on the AT properties has been assessed by comparing the AT between cohorts of individuals habitually exposed to different activities. Male runners were shown to have a significantly larger CSA [75], especially at the most distal part of the tendon (+36.0%) [74]; while the sprinters also had higher AT stiffness and maximal tendon forces than the endurance runners and non-active participants [54]. Furthermore, male athletes who frequently performed weight-bearing exercises (e.g., running, jumping) were found to have a larger AT CSA than athletes in non-weight-bearing sports (e.g., kayakers) [76]. This study reported that the increased AT CSA would be subjected to intermittent high-tension tendon loading. However, the effect of different training methods on tendon characteristics and injury risk needs further study. Another study found that the AT in the jump leg of male collegiate-level jumpers was much stiffer (17.8% and 24.4% greater stiffness and Young’s modulus, respectively) compared to the non-jump leg [77]. From the perspective of pathology, the increased AT stiffness can improve the transmission of muscle-generated forces [30,54] and reduce the risk of accumulating damage and performance failing [78]. Thus, compared to non-runners, habituated runners with high ankle plantar flexor strength may show improved adaptations to high mechanical loads during graded voluntary 10-s isometric plantarflexion efforts because of the increased AT stiffness, which would enable more direct transmission of muscle force [75].

Not surprisingly, the greater AT CSA, Young’s modulus, and stiffness of the individuals in the running, jumping, and sprinting events represent a favorable adaptation in response to the habitual loading of running. Regarding running, among the runners with different foot strike patterns (FSP: forefoot—FFS, midfoot—MFS, and rearfoot—RFS), Kernozek et al. [79] found habitual FFS/MFS runners did not have greater CSA despite higher AT loading according to a cross-sectional study that was conducted by recruiting female runners. In addition, Kubo et al. [47] reported no significant differences in the CSA and stiffness of AT by recruiting trained male long-distance participants. It is, however, noteworthy that the FSP during shod running was determined at a velocity of 18 km/h which was much higher than most of the other running studies. Being inconsistent with Kubo et al., a previous study [80] showed the AT in minimalist shod runners (MFS/FFS) adapted by increasing +9.2% CSA, +90.5% stiffness, and +89.8% Young’s modulus compared to traditionally shod runners (RFS), which seems more in accordance with the understanding of mechanical adaptation of the tendon to different AT loading patterns among runners with different FSP [81]. This inspired us that multiple exercise and training modalities combined with a step-by-step approach could lead to AT biomechanical adaptations, so may do minimalist running.

## 4. Conclusions

Firstly, high compliance of the AT was observed after the acute exercise intervention. Moreover, there are gender-specific effects in response to the acute loading. Specifically, the stiffness and Young’s modulus of the AT show significantly lower in women compared to men, which may be an important cause of fewer AT injuries in women. Secondly, periodic plyometric training can improve the morphological adaptability of the AT, but the exercise type, intensity, and duration are the three key factors that restrict the effectiveness of training. Finally, habitual running can effectively improve the mechanical properties of the AT (e.g., greater stiffness, CSA, and Young’s modulus). In particular, the high stiffness of the AT is conducive to enhancing the transmission of muscle strength, thereby improving the running economy. Future research is required to explore more training methods to improve the biomechanical adaptability of the AT from the perspective of mechanism and multiple influencing factors and reduce the risk of AT-related injuries.

## Figures and Tables

**Table 1:** Effects of plantar flexor acute exercise on the AT mechanical properties.

Study Information				Participants			Time of Assessment	Outcome Measure and Results
Authors	Design	Exercise Type	Exercise Protocol	N (M/W)	Age (Years)	Training Status		
Farris et al. [32]	Parallel	Running	A single 30-min at 12 kmph	12 (M)	27 ± 5	N/D	Before, immediately after the intervention	AT stiffness- andpeak AT strain-
Joseph et al. [31]	Cross	Jumping	Load equalling 20% body mass	31 (17M/14W)	24.1 ± 2	Active	Rest, immediately after intervention and a fatigue protocol	AT elongation↑, stiffness↓ and Young’s modulus ↓(W)
Kay & Blazevich [33]	Cross	MVIC	Passive and active concentric trials	16 (8M/8W)	20.2 ± 2.6	Active	Before, real-time, immediately after the intervention	AT stiffness↓
Maganaris et al. [34]	Cross	Cycling	Increasing load	6 (M)	23 ± 2	N/D	Before, immediately after each intervention	AT length-
Morse et al. [35]	Cross	Passive stretching	ROM at deg s^−1^ for 1 min	8 (M)	20.5 ± 0.9	Active	Before, immediately after the intervention	AT length-

Note: Cross, crossover design. MIVC, maximal isometric voluntary contraction. ROM, range of motion. M/W, men/women. N/D, not described. ↑/↓, significant increase/decrease. -, no significant difference.

**Table 2:** Effects of running training programs on the AT adaptation.

Author	Participants (*n*)	Age (Years)	Training Program	Instrument	Area of Measures	Outcome Measure and Results
Hansen et al. [56]	11 (7M/4W) untrained healthy individuals	29 ± 1 (M); 26 ± 1 (W)	About 9 months of habitual running	ultrasound; MRI	Triceps surae and AT	AT CSA-
Joseph et al. [57]	22 (7M/15W) traditionally shod runners	21.1 ± 3.3 (M); 22.4 ± 6.4 (W)	12-week transition with minimalist shoe running	Ultrasound;	AT	AT CSA↑; AT force↑, elongation↓ and stiffness↑; Young’s modulus↑
Milgrom et al. [58]	55 (M) new elite infantries	19.7 ± 0.8 (M)	6-month military training	Ultrasound;	AT	AT CSA↑
Zhang et al. [51]	17 (M) habitual recreational runners	30.6 ± 6.8 (M)	12-week transition training with the minimalist in habitual rearfoot strike runners	Ultrasound;	AT and ankle joint	AT force↑; AT CSA↑

Note: M/W, men/women. CSA, the cross-sectional area. N/D, not described. MRI, magnetic resonance imaging. ↑/↓, significant increase/decrease. -, no significant difference.

**Table 3:** The differences in the AT stiffness, Young’s modulus, CSA and strength between runners and non-runners.

Author	Participants (*n*)	Age (Years)	Test Methods	Dependent Variables			
				Stiffness	Young’s Modulus	CSA	Strength
Arampatzis et al. [54]	Non active (10); endurance runners (28); sprinters (28)	26 ± 5 (M);	Hold 2–3 s isometric MVC plantarflexion	Non active < endurance runners < sprinters *	N/D	N/D	Non active < endurance runners < sprinters *
Magnusson et al. [74]	Runners (6); non-runners (6)	36 ± 7 (M); 34 ± 3 (M)	MRI	N/D	N/D	Non-runners < runners *	N/D
Rosager et al. [75]	Runners (5); non-runners (5)	34 ± 6 (M); 33 ± 8 (M)	graded voluntary 10 s isometric plantarflexion	Non-runners < runners	Non-runners > runners	Non-runners < runners *	Non-runners < runners

Note: M/W, men/women. CSA, the cross-sectional area. N/D, not described. MRI, magnetic resonance imaging. *, statistically significant differences between runners or sprinters and other group(s).

## Data Availability

No applicable.
